# A Case of Isolated Primary Pleural Neurofibroma in a 39-Year-Old Woman

**DOI:** 10.1155/2019/6458302

**Published:** 2019-11-24

**Authors:** Kritika Krishnamurthy, John Alexis, Pukhraz Basra, Ana Maria Medina

**Affiliations:** ^1^AM Rywlin Department of Pathology and Laboratory Medicine, Mount Sinai Medical Centre, Miami Beach, FL, USA; ^2^Florida International University Herbert Wertheim College of Medicine, Miami, FL, USA

## Abstract

Primary benign neurogenic neoplasms of the pleura are exceedingly rare. Neurofibromas rarely involve the pleura. A review of the literarture reveals only a single reported case of isolated pleural neurofibroma. Herein the authors describe another case of isolated primary pleural neurofibroma. A 39-year-old nonsmoker woman presented to the emergency room with complaints of progressively worsening chest pain of one month duration. A computed tomography of the chest revealed a crescent shaped, pleural based mass suspicious for a neurogenic tumor such as an intercostal schwannoma. A PET-CT skull base to midthigh failed to reveal any other masses or abnormalities. A surgical excision of the mass was performed due to the patient's intractable pain. The resected specimen consisted of an ovoid fragment of soft tissue with pale yellow, smooth and glistening cut surface. Microscopic examination revealed the tumor to be composed of spindle cells with wavy nuclei arranged haphazardly in loose collagenous and pale myxoid stroma with rare interspersed mast cells. The spindle cells were diffusely positive for S100 protein and SOX-10, and focally positive for neurofilament. In the absence of any other masses in the patient and no pertinent history, a diagnosis of primary pleural neurofibroma was made. This case emphasizes the need to consider neurofibroma in any spindle cell neoplasm of the pleura irrespective of age or singularity.

## 1. Introduction

Pleural neoplasms are more commonly metastatic than primary. Primary neoplasms of the pleura are exceedingly rare [1]. They are more often malignant than benign, with malignant mesothelioma being the most common entity. Benign neoplasms include solitary fibrous tumor, lipomatous tumors, adenomatoid tumor, calcifying fibrous tumor, multicystic mesothelioma, and schwannoma [2]. Other benign tumor-like lesions include nodular pleural plaque and mesothelial cysts. Of these, solitary fibrous tumor is the most frequently encountered [3].

Neurofibromas are benign neurogenic tumors that rarely involve the pleura [2]. A review of the literarture reveals only a single reported case of isolated pleural neurofibroma. Herein the authors describe another case of isolated primary pleural neurofibroma.

## 2. Case Report

**Figure 1:**
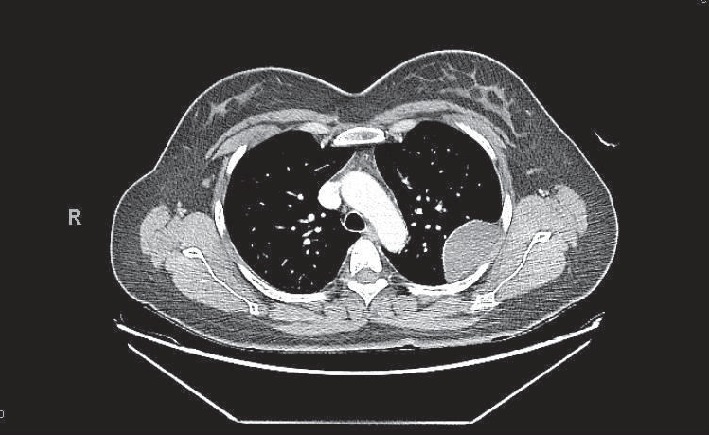
A crescent shaped, pleural based mass, measuring 6 × 4.9 × 3.8 cm, with some modelling and cortical irregularity of the superior aspect of the left lateral fourth rib as seen on CT.

The patient is a 39-year-old woman who presented to the emergency room with complaints of progressively worsening chest pain of one month duration. The pain was of gradual onset, moderate in intensity, constant, and localised to the left upper quadrant. She was nonsmoker with no significant medical history. Physical examination was within normal limits. A computed tomography of the chest revealed a crescent shaped, pleural based mass, measuring 6 × 4.9 × 3.8 cm, with some modelling and cortical irregularity of the superior aspect of the left lateral fourth rib suggestive of a long standing process (Figure 1). These imaging findings were suspicious for a neurogenic tumor such as an intercostal schwannoma, though other more aggressive etiologies could not be ruled out. A PET-CT skull base to midthigh failed to reveal any other masses or abnormalities. A CT guided biopsy of the mass was attempted, but the specimen was not diagnostic consisting only of scant adipose tissue.

A surgical excision of the mass was performed due to the patient's intractable pain. Intraoperatively, the pleural mass was ovoid, smooth, well-circumscribed and could be separated from the lung parenchyma. It was resected and sent to pathology.

**Figure 2:**
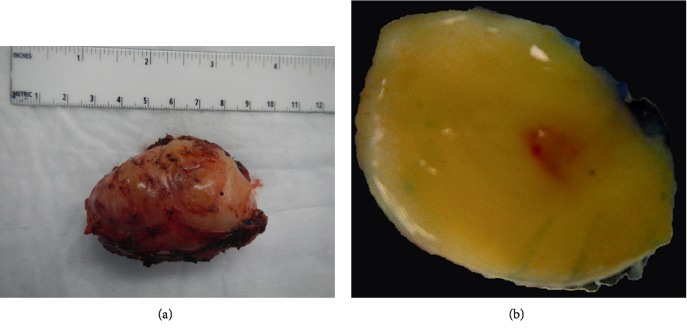
(a) Well circumscribed, ovoid mass. (b) Yellow, smooth and glistening cut surface.

**Figure 3:**
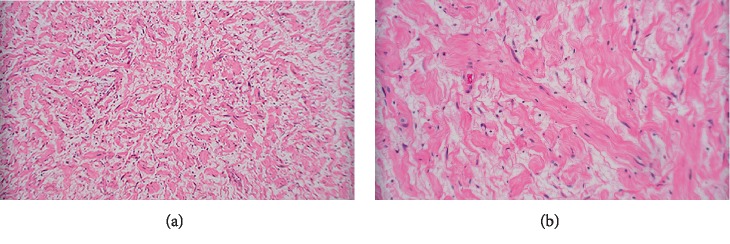
(a) Spindle cells arranged haphazardly in loose collagenous and pale myxoid stroma (100x). (b) Spindle cells with wavy nuclei (400x).

**Figure 4:**
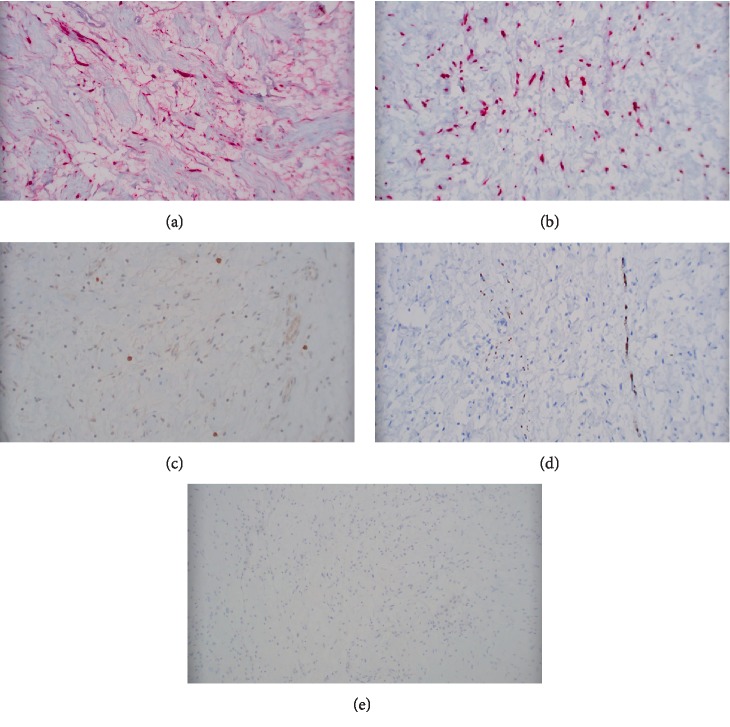
(a) Tumor cells diffusely positive for S100 protein (200x). (b) Tumor cells showing SOX-10 positivity (200x). (c) CD117 highlighting mast cells (200x). (d) Neurofilament highlighting the trapped axons (200x). (e) Tumor cells negative for STAT6 (100x).

The resected specimen consisted of an ovoid fragment of soft tissue measuring 7 × 5.3 × 2.4 cm. The cut surface was pale yellow, smooth and glistening (Figures 2(a) and 2(b)). Microscopic examination revealed the tumor to be composed of spindle cells with wavy nuclei arranged haphazardly in loose collagenous and pale myxoid stroma with rare interspersed mast cells (Figures 3(a) and 3(b)). This histopathological picture was suggestive of neurofibroma. On immunohistochemistry, the spindle cells were diffusely positive for S100 protein and SOX-10, and focally positive for neurofilament. CD34 showed some fibrillary positivity while STAT-6 and bcl-2 were negative. CD117 highlighted the mast cells (Figures 4(a)–4(e)). In the absence of any other masses in the patient and no pertinent history, a diagnosis of primary pleural neurofibroma was made.

The patient's post-surgical course was uneventful and continues to be in follow-up with no new complaints at the time of this report.

## 3. Discussion

Primary benign pleural neoplasms are infrequent [2]. However, it is important to differentiate these uncommon tumors from the more commonly encountered metastatic cancers and malignant mesothelioma as prognosis and management are radically different.

Due to the extensive nerve supply throughout the thorax, neurogenic tumors arise in this location and account for approximately 9% of primary mediastinal tumors in adults [4]. Ribet et al. conducted a retrospective review of 134 cases of neurogenic tumors of the thorax and found them to be benign in 93% of adult cases with nerve sheath tumors comprising 73.5% of adult cases [5].

Benign nerve sheath tumors encountered in the thorax include schwannomas, ganglioneuromas, and neurofibromas [6]. Ratbi et al. studied 17 patients who underwent resection of mediastinal benign neurogenic tumors and found schwannoma in 76.4% cases [7]. The rarity of intrathoracic neurofibromas was further confirmed in a study of 149 intrathoracic neurogenic tumors by Bicakcioglu et al. [8].

Neurofibromas originate from Schwann cells and fibroblasts and may arise in any peripheral nerve. They most often affect patients between 20 and 30 years of age and have no sex predilection [9]. Approximately 90% of the neurofibromas are localised and not associated with neurofibromatosis [10]. Localised neurofibromas tend to be slow growing and asymptomatic, although they may expand and cause symptoms due to compression of nerves or other adjacent structures [11]. In our case the patient had a relatively large tumor that may have been compressing adjacent nerves resulting in pain. Gupta et al. described a well-defined homogeneous isodense and nonenhancing mass with rib abnormalities due to bone erosion from the adjacent neurofibroma, similar to the radiologic findings seen in our case [12].

Pleural involvement by neurofibroma is exceedingly rare with only three cases reported so far [12, 13]. Of these, only one case reported by Langman et al., was not associated with neurofibromatosis [13]. Primary pleural neurofibromas arise directly from intercostal nerves and form pleural based masses. The only case of isolated primary pleural neurofibroma is a 71 year old woman who presented with shoulder pain, reported from Europe. Our case is the first reported case of isolated primary pleural neurofibroma in the United States. Though our patient presented with similar complaints of pain as the previous reported case, her younger age of presentation sets her apart.

The differential diagnosis for neurofibromas in the pleura includes but is not limited to neural neoplasms, such as schwannoma and ganglioneuroma, as well as a variety of nonnerve sheath tumors, in particular solitary fibrous tumor, mesothelioma, synovial sarcoma, fibrosarcoma, and undifferentiated pleomorphic sarcoma. The malignant neoplasms such as mesothelioma, synovial sarcoma, fibrosarcoma, and undifferentiated pleomorphic sarcoma can be excluded due to the absence of any features of malignancy in conventional neurofibromas while benign neoplasms such as ganglioneuroma have characteristic histologic features that preclude confusion with neurofibroma.

Differentiating a neurofibroma from a schwannoma and solitary fibrous tumor may sometimes be challenging. The presence of mast cells, which is integral in the development of neurofibromas, is one of the most specific hisopathological features that help clinch the diagnosis of neurofibroma [14, 15]. Immunohistochemistry may also be helpful. Neurofibromas are invariably positive for S-100 protein and negative for STAT6 and bcl-2, in contrast to solitary fibrous tumors which are negative for S-100 protein and positive for STAT6 and bcl-2 [16–18]. In our case, the tumor cells are positive for S-100 protein and negative for bcl-2 and STAT 6, ruling out solitary fibrous tumor. Schwannomas, like neurofibromas, are positive for S-100 protein and SOX-10, and may show focal CD 34 positivity while being bcl-2 negative, thus making them harder to discern from neurofibromas [17, 19]. Localisation of staining for neurofilament protein may be of some value in differentiating the two. Neurofibromas grow within the nerve of origin; accordingly neurofilament protein will stain axons entrapped within the tumor. Whereas, schwannomas grow peripherally and displace the nerve of origin, hence staining for neurofilament protein will fail to reveal any entrapped axons within the tumor though occasionally axons may be entrapped at the very periphery [20]. However, some recent studies suggest that intralesional axons may be more frequently seen in schwannomas than previously reported [21].

Treatment of localized neurofibromas is complete surgical resection. Neurofibromas grow within and cannot be separated from normal nerve, and complete excision of the neoplasm requires sacrifice of the nerve. This is acceptable in cases of pleural neurofibroma as they do not involve major nerves. Local recurrence after complete excision is unusual [22].

## 4. Conclusion

This case emphasizes the need to consider neurofibroma in any spindle cell neoplasm of the pleura irrespective of age or singularity. The main differential diagnosis includes schwannomas and solitary fibrous tumor which can be excluded in most cases by histopathology supplemented with immunohistochemistry. Complete surgical resection is curative.
